# Synthesis and Application Studies of DOPO-Based Organophosphorous Derivatives to Modify the Thermal Behavior of Polybenzoxazine

**DOI:** 10.3390/polym14030606

**Published:** 2022-02-03

**Authors:** Thorben Sören Haubold, Andreas Hartwig, Katharina Koschek

**Affiliations:** 1 Fraunhofer Institute for Manufacturing Technology and Advanced Materials IFAM, Wiener Strasse 12, 28359 Bremen, Germany; Thorben.Haubold@ifam.fraunhofer.de (T.S.H.); Andreas.Hartwig@ifam.fraunhofer.de (A.H.); 2 Department 2 Biology/Chemistry, University of Bremen, Leobener Strasse 7, 28359 Bremen, Germany

**Keywords:** UL-94 test, halogen-free flame retardant, benzoxazine, DOPO

## Abstract

The DOPO-based flame-retardant additives DOPO-HQ, DOPO-AP and DOPO-Van were synthesized in varying numbers of phenolic hydroxyl groups and amine groups. Moreover, their influence on the polymerization of a bisphenol F-based benzoxazine, as well as the thermal properties of the resulting materials, were studied. All DOPO-based derivatives influenced the polymerization temperature onset with a reduction of up to 20 °C, while thermo-mechanical properties remained high. Surprisingly, phosphorous content below 0.4 wt% significantly improved the reaction against small flames yielding an increase in the limited oxygen index by 2% and a V-0 rating in the UL-94 test. DOPO-HQ proved to be the most effective additive regarding the reaction against small flames at an astonishingly low phosphorous concentration of below 0.1 wt%, whereas DOPO-AP and DOPO-Van simultaneously lowered the polymerization temperature.

## 1. Introduction

Polybenzoxazines (pBz) represent a phenolic-type thermoset, and exhibit excellent properties such as mechanical performance, chemical stability, low water absorption [1], a near zero volume shrinkage [2], and polymerize by-product free [3]. High polymerization temperatures limit their broad application. Bifunctional catalysts [4], lewis acids [5], tosylated aliphatic alcohols [6], imidazoles [7], or carboxylic acids [8,9] have been investigated, showing promising results regarding a lowering effect on the onset and peak temperature of the ring-opening polymerization (ROP). Additionally, it has been shown that phenols accelerate ring-opening polymerization due to their acidic protons that protonate either the oxygen or nitrogen atom of the oxazine ring, starting the ROP at lower temperatures [9,10,11,12,13]. Amines react with benzoxazine monomers over several steps. First, the amine attacks the benzoxazine, yielding a zwitter-ionic product that decomposes to an iminium ion that reacts in an electrophilic substitution reaction with the aromatic ring [14,15].

From an application point of view, pBz-based materials do not fully fulfil flame retardancy requirements for certain applications, e.g., in electrical applications for circuit boards [16], despite their good thermal properties. Basically, flame retardancy of polymeric materials can be improved by incorporating different flame retardant (FR) relevant groups, e.g., halogens [17], organo-phosphorous [18], or silicium-based groups [19], into the polymeric network. Depending on the functional group, different mechanisms of flame retardancy can be addressed—namely, thermal shielding in the solid phase, dilution of the gas phase, quenching of radicals in the gas phase or endothermic degradation. FR groups can be incorporated by monomer design [20], or by addition of reactive [21] as well as non-reactive FR additives [22].

A variety of benzoxazine monomers containing flame-retardant relevant groups has been published. Li et al. have introduced bromine in different benzoxazine monomers and achieved a V-0 rating in UL-94 tests for the resulting pBz [17,23]. However, halogen-containing polymers release toxic gasses during fire, and are therefore of diminishing interest for industrial applications. Organophosphorous-based benzoxazine monomers have been described beside others by Choi et al. [18], Zuniga et al. [24], Chang et al. [25], and Lin et al. [20,26,27]. In comparison to halogens, phosphorus-based FRs do not release toxic volatiles. Instead, they cover the outer layer of the polymer with a non-flammable coating, or release phosphorous oxide radicals with a radical trapping ability during combustion. FR groups incorporated into benzoxazine monomers by molecular design result in polybenzoxazines with good mechanical properties. They do not alter the polymerization process significantly. Thus, polymerization temperatures remain high.

Both challenges—high polymerization temperature and flame retardancy—can be approached with the right choice of reactive or non-reactive additives. Benzoxazine monomers have been polymerized in the presence of additives such as nitrogen [28,29], silicon [30], and organophosphorous-based additives [27,31], that improved flame retardancy and reduced the polymerization onset simultaneously. However, most of these reduced the glass transition temperature (*T*_g_), and thus affected the thermo-mechanical properties.

The reactive additive DOPO (9,10-dihydro-9-oxa-10-phosphaphenanthrene-10-oxide) and its derivatives bearing hydroxyl groups have proven to be potential candidates to address this double challenge. DOPO is mainly gas-phase active, yielding PO and HPO upon decomposition that trap energetic radicals [32]. Lin et al. studied the effect of DOPO and the three-functional phenolic DOPO-based additive dopotriol on a bisphenol F and aniline-based benzoxazine (BF-a) [27]. Neat DOPO lowered the polymerization temperature to such an extent that polymerization started upon mixing. The resulting polymer showed significant improvement regarding the UL-94 test yielding a V-0 rating at a phosphorous content of 1 wt%. Moreover, it showed a weak to moderate effect on the glass transition temperature (*T*_g_), with a reduction of 11 °C. In contrast, dopotriol affected the polymerization onset temperature (*T*_onset_) to a lesser degree. A V-0 rating was achieved with a phosphorous content of solely 0.5 wt%, without affecting the *T*_g_.

Taylor et al. studied DOPO with a bisphenol A and aniline-based benzoxazine monomer (BA-a) and compared it to the reactive FRs bis(4-hydroxyphenyl)phenylphosphine oxide (BHPPO) and diphenyl phosphoramidate (DPPA) [31]. In contrast to pBF-a-based systems, 10 wt% DOPO increased the *T*_g_, while still reducing the *T*_onset_ significantly. Comparable improvements were achieved with BHPPO and DPPA. Regarding the thermal stability, DOPO changed the decomposition behavior as it strongly affected char formation. However, the char at 800 °C was less stable, since the char yield decreased in comparison to neat pBA-a. BHPPO and DPPA did not affect the decomposition behavior significantly, and increased the char slightly at 10 wt% for both additives. Thus, the DOPO and hydroxyl groups containing DOPO derivatives showed a beneficial effect on the flame retardancy and polymerization temperature of BF-a- and BA-a-based polybenzoxazines.

**Figure 1:**
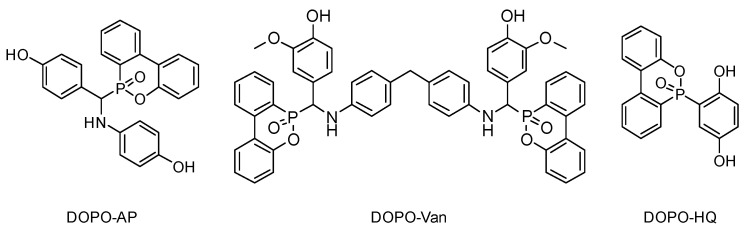
Chemical structures of the reactive additives DOPO-AP, DOPO-Van and DOPO-HQ.

In the current state of our knowledge, a systematic study of DOPO as an FR functional group differing in type and number of reactive groups affecting benzoxazine polymerization is missing thus far. This contribution aims at investigating the influence of additives combining both hydroxyl and amine groups by lowering the polymerization temperature and DOPO moieties providing lower flammability. For this purpose, three different DOPO-based additives, with at least two phenolic hydroxyl groups each, were synthesized and investigated (Figure 1). DOPO-HQ [33,34], DOPO-Van [35] and DOPO-AP [36,37] have two hydroxyl groups able to initiate and influence the polymerization reaction. Additionally, DOPO-AP exhibits one, and DOPO-Van two, secondary amine groups. The secondary amine groups are expected to influence *T*_onset_, whereas the impact should be less intense compared with the phenolic hydroxyl groups. Moreover, the additives differ in the number of DOPO groups, with DOPO-HQ and DOPO-AP bearing one, and DOPO-Van bearing two DOPO groups as the FR motif, respectively. Thereby, the phosphorous weight percentage (Pwt%) in the resulting polybenzoxazines vary appropriately.

Those additives were mixed in low concentrations with BF-a monomer aiming at a systematic investigation of the impact on the polymerization onset, reaction against small flames and thermo-mechanical properties. With this approach, low concentrations of halogen-free and reactivity-enhancing additives could represent a sustainable approach by saving resources and energy during polybenzoxazine manufacturing and application.

## 2. Materials and Methods

### 2.1. Materials

Bisphenol-F and aniline-based benzoxazine Araldite MT35700^®^ (BF-a) was obtained from Huntsman Corporation (Basel, Switzerland), and 4,4′-methylenedianiline (MDA) from Fluka (Buchs, Switzerland). *p*-Aminophenol, *p*-benzoquinone, ethanol, 2-ethoxyethanol, methanol, tetrahydrofuran and vanillin were supplied from Sigma Aldrich (Taufkirchen, Germany). 4-hydroxybenzaldehyde was from ABCR (Karlsruhe, Germany), and DOPO was from TCI (Zwijndrecht, Belgium). All chemicals were used without further purification.

### 2.2. Synthesis

#### 2.2.1. Synthesis of 6,6′-(((Methylenebis(4,1-phenylene))bis(azanediyl))bis((4-hydroxy-3-methoxyphenyl)methylene))bis(6H-dibenzo(c,e)(1,2)oxaphosphine 6-oxide (DOPO-Van)

The synthesis was performed according to Gu et al. [35]. MDA (1.49 g, 7.52 mmol) was dissolved in ethanol (15 mL) in a 100 mL two-neck round-bottom flask. After complete dissolution, a vanillin (2.28 g, 15.0 mmol) solution in ethanol (20 mL) was added with a dropping funnel over the course of 30 min. The reaction mixture was heated at 40 °C for 4 h. DOPO (3.24 g, 15.0 mmol) was added as powder over the course of 10 min. Ethanol (20 mL) was added to prevent precipitation. The reaction mixture was heated to 80 °C for 14 h, and decanted after cooling to 25 °C. The crude product was recrystallized in ethanol (25 mL), filtered, and dried under vacuum at 60 °C for 2 h. After grinding and further drying at 80 °C under vacuum for 12 h, a yellowish powder was received with a yield of 66%.

^1^H NMR (600 MHz, DMSO-*d*_6_), δ = 8.92-8.88 (d, 2H), 8.20-8.12 (m, 4H), 7.87 (dd, *J* = 9.8, 6.0 Hz,1H), 7.76-7.66 (m, 3H), 7.55-7.49 (m, 1H), 7.45-7.36 (m, 3H), 7.29 (q, *J* = 7.3, 6.8 Hz, 2H), 7.13 (d, *J* = 8.1 Hz, 1H), 7.04 (d, *J* = 8.1 Hz, 1H), 6.93-6.87 (m, 2H), 6.78-6.47 (m, 10H), 6.43-6.34 (m, 1H), 6.05-6.02 (m, 1H), 5.26-5.18 (m, 1H), 4.90-4.82 (m, 1H) 3.65 (s, 3H), 3.62 (s, 3H), 3.42 (m, 2H) ppm.

^31^P NMR (243 MHz, DMSO-*d*_6_) d 31.56 (s), 28.84 (s) ppm.

#### 2.2.2. Synthesis of 2-(6-Oxido-6H-dibenz(c,e)(1,2)oxaphosphorin-6-yl)-1,4-hydroxy phenylene (DOPO-HQ)

The synthesis was adjusted in accordance with Ho et al. [34] and Wang et al. [33]. DOPO (5.40 g, 25.0 mmol) and 2-ethoxyethanol (20 mL) were mixed and heated to 90 °C. After complete dissolution, *p*-benzoquinone (2.43 g, 22.5 mmol) was added as a powder over the course of 10 min and the reaction mixture was heated to 125 °C for 4 h. The mixture was filtrated after cooling to 25 °C, and the powder was recrystallized in 2-ethoxyethanol. The product was dried in a vacuum oven at 90 °C for 12 h to obtain an off-white powder in 73% yield.

^1^H NMR (600 MHz, DMSO-*d*_6_), δ = 9.46 (s, 1H), 9.17 (s, 1H), 8.27-8.20 (m, 2H), 7.74 (t, *J* = 7.7 Hz, 2H), 7.55-7.62 (dd, *J* = 13.6, 6.9 Hz, 1H), 7.50 (dt, *J* = 7.5, 2.0 Hz, 1H), 7.45 (t, *J* = 7.7, 1 H), 7.32-7.26 (m, 2H), 7.19 (dd, *J* = 15.2, 3.1 Hz, 1H), 6.89 (dd, *J* = 8.7, 3.1 Hz, 1H), 6.64 (dd, *J* = 8.6, 7.5 Hz, 1H) ppm.

^31^P NMR (243 MHz, DMSO-*d*_6_) δ = 22.11 (s) ppm.

#### 2.2.3. Synthesis of 4-(2′-Hydroxybenzylidene imino)phenol (**1**)

The synthesis was performed according to Lin et al. [36]. 4-Aminophenol (8.94 g, 81.9 mmol) and 4-hydroxybenzaldehyde (10.0 g, 82.0 mmol) were dissolved in methanol (50 mL) in a two-neck round-bottom flask. The mixture was stirred under nitrogen atmosphere for 5 h at 50 °C. Methanol (10 mL) was added and the product was precipitated by pouring in water. The crude product was washed with cold methanol and then dried in a vacuum oven for 12 h at 50 °C. The product was received as white powder in 85% yield.

^1^H NMR (600 MHz, DMSO-*d*_6_), δ = 9.86 (s, 1H), 9.48 (s, 1H), 8.43 (s, 1H), 7.72 (d, *J* = 8.5 Hz, 2H), 7.12 (d, *J* = 8.7 Hz, 2H), 6.81 (dd, *J* = 8.6, 7.2 Hz, 4H) ppm.

#### 2.2.4. Synthesis of 4-((4′-Hydroxyphenylimino)(6-oxido-6h dibenz(c,e)(1,2)oxaphosphorin-6-yl)methyl)phenol (DOPO-AP)

The synthesis was adapted according to Xiong et al. [37]. DOPO (29.85 g, 138.1 mmol) and **1** (14.7 g, 69.0 mmol) were dissolved in THF (100 mL) in a two-neck round-bottom flask with a reflux condenser. The resulting solution was heated to 60 °C for 12 h under N_2_ atmosphere. The mixture was cooled to 25 °C and the precipitate was filtrated and then washed with cold THF. The product was dried in a vacuum oven for 12 h at 75 °C and was obtained in 61% yield.

^1^H NMR (600 MHz, DMSO-*d*_6_), δ = 9.38, 9.34′ (s, 1H), 8.43, 8.45′ (s, 1H), 8.22-8.16, 8.22-8.16′ (m, 2H), 8.03, 7.69′ (dd, *J* = 10.5, 7.7 Hz, 1H), 7.77, 7.73′ (td, *J* = 7.8, 1.4 Hz, 1H), 7.56, 7.44′(td, *J* = 7.5, 2.4 Hz, 1H), 7.45, 7.45′ (t, *J* = 7.1 Hz, 1H), 7.16-7.13, 7.19-7.16′ (m, 1H), 7.03, 7.29′ (dd, *J* = 8.1, 1.2 Hz, 1H), 6.67, 6.62′ (d, *J* = 8.5 Hz, 2H), 6.46, 6.51′ (d, *J* = 8.9 Hz, 2H), 6.37, 6.40′ (d, *J* = 8.9 Hz, 2H), 5.96, 5.53′ (dd, *J* = 11.0, 4.9 Hz,1H), 4.78, 5.19′ (dd, *J* = 16.4, 11.0 Hz, 1H) ppm.

^31^P NMR (243 MHz, DMSO-*d*_6_), δ = 31.79′ (s), 28.80 (s) ppm.

### 2.3. Sample Preparation and Polymerization Protocol

Powdered BF-a and the appropriate FR additive were mixed and melted in a ventilated oven for 20 min at 140 °C, before further mixing in a planetary centrifugal mixer under vacuum. The homogeneous melt was either used for further analysis or cast in a metal mold (170 × 130 × 4 mm^3^). The mold was transferred into a vacuum oven and was degassed for 30 min at 140 °C. Afterwards the melt was cured with the following temperature profile 170 °C for 2 h, 180 °C for 2 h and then 200 °C for 2 h. The specimen were demolded and cut according to specimen dimensions for DMA, LOI and UL-94 testing.

### 2.4. Equipment and Characterization

FT-IR spectra were recorded on an Equinox 55 ATR spectrometer (Bruker, Bremen, Germany) in attenuated total reflection (ATR) mode using a diamond crystal with 32 scans and a resolution of 4 cm^−1^. The range was from 500 to 4000 cm^−1^.

^1^H NMR spectra were recorded on an AVANCE NEO 600 MHz NMR-spectrometer (Bruker, Bremen, Germany) with DMSO-*d*_6_ as solvent at 20 °C. ^31^P NMR spectra were recorded in 243 MHz using the same spectrometer. The chemical shifts were reported in parts per million (ppm) and referred to residual protons in the DMSO-*d*_6_.

DSC measurements were performed using a Discovery DSC (TA Instruments, Hüllhorst, Germany) with 10 K/min in a range from 0 °C to 300 °C on a specimen scale of 1–2 mg under N_2_ atmosphere. All samples were measured 3 times and the average was taken for evaluation. The *T*_onset_ was determined as a derivative of heat flow and temperature curve using the TRIOS software.

DMA experiments were performed using the Dynamic Mechanical Analyzer Q800 (TA Instruments, Hüllhorst, Germany). Thermo-mechanical properties were determined in a temperature range from 0 °C to 250 °C, with a heating rate of 2 K/min, and a frequency of 1 Hz in a single cantilever bending mode, using a test specimen of 40 × 10 × 4 mm^3^. Glass transition temperature (*T*_g_) was determined by the maximum of loss modulus.

TGA measurements were carried out with the Q5000 (TA Instruments, Hüllhorst, Germany) in a temperature range from 25 °C to 800 °C, and a heating rate of 10 K/min under ambient and nitrogen atmosphere. In addition, 5, 15, 25 and 40 K/min were additional heating rates applied for experiments under nitrogen atmosphere. Mass loss derivatives were calculated from the TGA results.

UL-94 is an international standard test released by Underwriters Laboratories (UL) (Northbrook, IL, USA) to determine the tendency of a polymeric material to extinguish or spread flames upon ignition. UL-94 tests were performed according to DIN EN 60695-11-10 with specimen dimensions of 125 × 13 × 4 mm^3^ and grinded edges. Samples were treated two times with an open flame for 10 s, measuring the time T_1_ and T_2_, respectively, until the samples extinguished themselves after removing the flame. The total burning time (T_1_ and T_2_) was used for classification.

The Limited Oxygen Index (LOI) determines the needed oxygen concentration for the combustion of a polymeric material. LOI experiments were performed according to EN ISO 4589-2 with specimen type 1, dimensions of 100 × 10 × 4 mm^3^ and grinded edges with an LOI apparatus (East Grinstead, UK). The LOI value was determined as followed:

(1) $$\mathrm{LOI}=c_{F}+k\cdot d$$

where $c_{F}$ is the final value of the oxygen concentration, *d* is the step size between oxygen concentrations, and *k* is a factor determined by the results of the test specimen. The standard deviation as error was calculated from:

(2) $$\sigma ={\left[\frac{\sum^{\;}{\left(C_{i}-\mathrm{LOI}\right)}^{2}}{n-1}\right]}^{\frac{1}{2}}$$

where *c*_i_ represents each of the last 6 determined oxygen concentrations, LOI, the determined limited oxygen index value, and *n*, the number of measurements of oxygen concentrations for *c*_i_.

## 3. Results and Discussion

### 3.1. Effect on the Polymerization Progress and Thermo-Mechanical Behaviour

**Figure 2:**
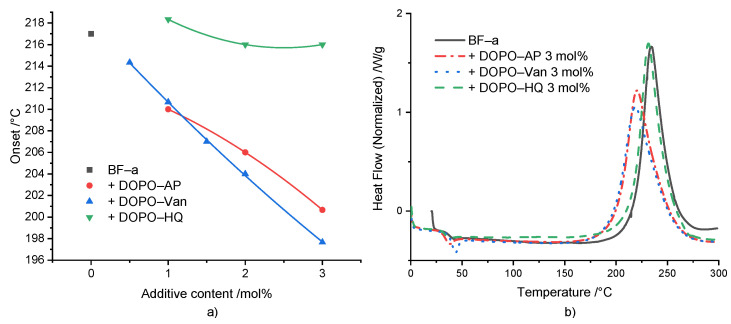
(**a**) Relation between polymerization onset and reactive additive content (the non-linear curve fit serves for visualization); (**b**) DSC comparison between BF-a and reactive mixtures with 3 mol% of FR additives.

ROP polymerization of BF-a in the presence of different concentrations of the DOPO-based additives DOPO-HQ, DOPO-Van and DOPO-AP was studied by DSC experiments (Figure 2a). For all additives, a concentration-dependent effect on the *T*_onset_ was displayed for the investigated concentrations ranging from 0.5 to 3 mol%. However, the additives showed a significant difference regarding the effectivity. In comparison to the neat BF-a with a *T*_onset_ = 218 °C, DOPO-HQ had the lowest impact with no effect at 1 mol%, and a slight shift to 216 °C at 3 mol%. This low impact was attributed to the sterical hindrance of a hydroxyl group caused by the bulky DOPO-group. Beside others, Wang et al. [33] and Pospiech et al. [38] showed that the bulky DOPO group sterically influenced the reaction products of DOPO-HQ upon etherification. Furthermore, as one hydroxyl group reacted, the sterical hindrance for the second one increased. DOPO-AP showed a stronger effect lowering the *T*_onset_ from BF-a from 218 °C to 210 °C at 1 mol%, and further to 201 °C at 3 mol%. The secondary amine group bridging the aromatic rings could initiate the polymerization. The same is true for the hydroxyl groups, even though the one in *para* position to the electron-donating amine group exhibited a slightly reduced acidity [39]. However, both hydroxyl groups were not sterically hindered. Thus, the amine and hydroxyl groups can be considered as reactive groups, which could initiate benzoxazines ROP. DOPO-Van showed the strongest influence decreasing *T*_onset_ from 214 °C at 0.5 mol% down to 198 °C at 3 mol%, with increasing additive concentration. It contained two amine groups and two phenolic hydroxyl groups. The presence of the methoxy group in *ortho* position should have caused an acidity of the hydroxyl group similar to the one for the hydroxyl group in *para* position in the methyl-substituted phenol of DOPO-AP [39,40]. The investigation on the effect of the substitution pattern of the phenolic group was according to the results of the substituted benzoxazine rings and their influence on the ROP [41,42,43].

For all formulations, an increase in enthalpy up to 30 J/g (ΔH = 280 J/g for BF-a) was observed, indicating an increased rate of conversion (Supplementary Table S1). In contrast to the results described by Taylor et al. [31] the reaction enthalpy did not show a consistent increase as a function of the different concentrations and different additives.

Figure 2b shows the DSC curves of neat BF-a and formulations with 3 mol% of the respective additive. In comparison to the neat BF-a, all additives did not influence the shape of the polymerization peak, but caused a distinguishable shift of the peak maximum towards a lower polymerization temperature. Additionally, DOPO-AP and DOPO-Van caused a slight broadening of the polymerization peak. 

**Figure 3:**
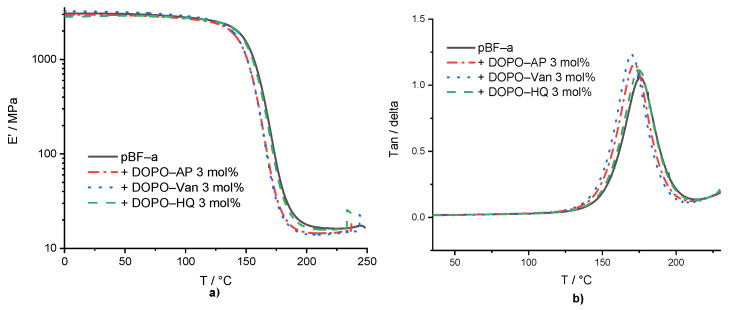
(**a**) Storage modulus of pBF-a and mixtures with 3 mol% FR additive and (**b**) tan (δ) of pBF-a and mixtures with 3 mol% FR additive.

Dynamic mechanical analysis (DMA) experiments were conducted in the temperature range of 0 °C to 250 °C, with pBF-a containing 3 mol% of each additive (Supplementary Table S2 and Figure 3). The aim was to study the impact of additive incorporation into the benzoxazines network on polymer network density and thermo-mechanical properties. Basically, the results proved that all additives did not significantly affect the thermo-mechanical behavior. Especially, the storage modulus of neat pBF-a (E’ = 2.8 GPa) nearly did not change in the presence of DOPO-Van: E’ = 2.9 GPa, DOPO-HQ: E’ = 2.8 GPa, and DOPO-AP: E’ = 2.7 GPa.

The *T*_g_ was determined by the maximum of loss modulus. The *T*_g_ of 157 °C for pBF-a as well as DOPO-HQ containing pBF-a indicated that the smallest molecule lacking amine groups did not significantly impact the polymer network density (Figure 3b), whereas DOPO-AP with increasing size reduced the *T*_g_ to 153 °C and DOPO-Van to 151 °C. Despite of the observed changes in the *T*_onset_ during benzoxazine polymerization in the presence of the DOPO additives, potential changes in polymer network topology did not affect the thermo-mechanical properties of the resulting pBF-a.

### 3.2. Thermal Stability

The aim of thermogravimetric analyses (TGA) was to assess the influence of additives on the decomposition behavior of pBF-a. As previously reported, for conventional polybenzoxazines the first degradation step is the cleavage of the Mannich bridge at around 300 °C, followed by the break-up of the bisphenol F backbone as a second degradation step around 380–420 °C [44,45]. Finally, the decomposition leading to the formation of the aromatic char as a third step takes place at 460 °C [46].

**Figure 4:**
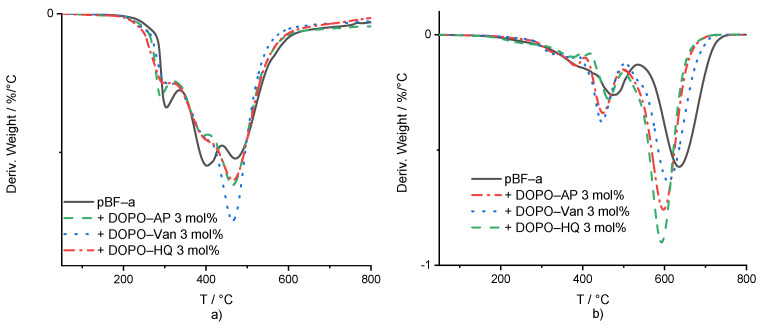
(**a**) First derivative of the mass loss curve in dependence of temperature for pBF-a and 3 mol% FR mixtures. (**b**) Mass loss derivative of thermo-oxidative decomposition against temperature for pBF-a and mixtures with 3 mol% FR.

Under the nitrogen atmosphere, DOPO-based additives did not significantly change the pBF-a’s decomposition behavior (Figure 4a and Table 1). The maximum peak temperatures of the weight loss derivatives were used to compare the degradation process in dependence of the present FR additive and its concentration. Regarding the first and second degradation steps, DOPO-AP and DOPO-Van did not change the temperature, while DOPO-HQ decreased both peak temperatures of the decomposition steps by around 10 °C to 292 °C and 394 °C, respectively. For all additives, the residual mass at the first and second corresponding peak temperature was around the same value as for pBF-a, with 96%. pBF-a mixture containing 3 mol% of DOPO-HQ, DOPO-AP or DOPO-HQ additives reduced the peak temperature for the third decomposition step slightly, from 472 °C to 468 °C, 467 °C and 464 °C, respectively. Furthermore, for all polymerized reactive mixtures, the mass loss ratio of the integrals of the second and third decomposition peaks changed in comparison to neat pBF-a with a decrease in the second degradation step and an increase in the third one. This indicates that all DOPO additives reduced the decomposition of the bisphenol F backbone and increased char formation. All 3 mol% FR-containing mixtures resulted a char yield between 45–46% at 800 °C, in comparison with a char yield of 44% for neat pBF-a. From this, it can be concluded that they did not impact the stability of the formed char. This observation is according to the influence of neat DOPO in BA-a [35].

In comparison with thermal decomposition, thermo-oxidative conditions for all additive-containing mixtures showed a decrease in the temperature of the first degradation step from 396 °C for pBF-a, to 378 °C, 381 °C and 385 °C for DOPO-Van, DOPO-AP and DOPO-HQ, respectively (Figure 4b and Supplementary Table S4). For the second degradation step, the peak temperatures for pBF-a mixtures were decreased from 481 °C with 3 mol% of DOPO-AP to 455 °C, with 3 mol% of DOPO-Van to 456 °C, and with 3 mol% of DOPO-HQ to 462 °C. While the peak temperatures were lower, DOPO-AP-, DOPO-Van- and DOPO-HQ-containing mixtures increased the residual mass at the peak temperature from 71% to 79%, 77% and 80%, respectively. The third peak in the mass loss derivative was 633 °C for neat pBF-a, and mixtures containing DOPO-HQ, DOPO-AP and DOPO-Van reduced to 597 °C, 603 °C and 614 °C, respectively. The residual mass at the maximum peak temperatures did not change. All formulations led to no residual mass at 800 °C. According to the literature, DOPO is considered to be mainly active in the gas phase [32].

**Figure 5:**
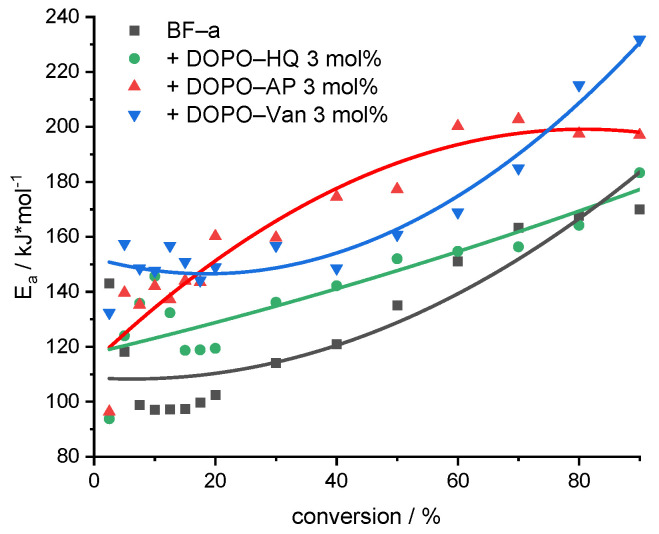
Evolution of the *E*_a_ with increasing conversion for the decomposition of pBF-a and samples containing 3 mol% of FR additive. The non-linear curve fit serves for visualization.

The efficiency of the flame retardancy is closely related to the temperature and the activation energy (*E*_a_) at which the FR volatile scavengers are produced. Degradation temperatures of the DOPO derivatives almost match the ones of pBF-a, or are slightly reduced in the case of DOPO-HQ. The *E*_a_ of the degradation is the energy needed for breaking or rearranging of chemical bonds. To obtain *E*_a_, TGA experiments were performed with different heating regimes under nitrogen atmosphere, and the Flynn–Wall–Ozawa (FWO) method [47] was used for the calculations for pBF-a and reactive mixtures with 3 mol% of DOPO-AP, DOPO-Van and DOPO-HQ. The FWO method is an isoconversional method that does not depend on the conversion function [48]. As long as the degradation mechanism stays the same for the investigated area, the method does not change with different heating rates for different conversions. The FWO method can be expressed by the following Equation (3):

(3) $$log_{10}\left(\beta \right)=log_{10}\left(\frac{AE_{a}}{R\left(\alpha \right)}\right)-2.315-0.4567\frac{E_{a}}{RT_{c}}\;$$

where β is the heating rate, *T_c_* in K is the temperature at a conversion α, with *A* as frequency factor, *R* as the gas constant, and *E*_a_ in J⋅mol^−1^ as the activation energy for the thermal degradation. The study was performed by using heating rates of 5, 10, 15, 25 and 40 K/min for pBF-a and reactive additive-containing mixtures (Supplementary Figure S7). The investigated conversions (α) for each heating rate and polymer were 2.5, 5, 7.5, 10 12.5, 15, 17.5, 20, 30, 40, 50, 60, 70, 80 and 90%. The *E*_a_ (determined by −0.4567$\frac{E_{a}}{RT}$) can be obtained from the slope of $log_{10}\left(\beta \right)$ versus 1000/*T*_c_ for different conversions from TGA data (Figure 5 and Supplementary Figure S8).

All investigated samples showed an increase in *E*_a_ with progressive conversion; they differed, however, in the absolute values (Figure 5). A comparison of the average *E*_a_ values revealed that DOPO-Van (*E*_a_ = 163 kJ/mol) and DOPO-AP (*E*_a_ = 160 kJ/mol) significantly improved the *E*_a_, whereas DOPO-HQ (*E*_a_ = 138 kJ/mol) showed a moderate effect (pBF-a *E*_a_ = 125 kJ/mol). Basically, all additives resulted in an increase in the initial stability, as the *E*_a_ value at 10% conversion corresponding to the first decomposition peak was higher in comparison to the neat pBF-a (*E*_a_ = 97 kJ/mol) for DOPO-AP (*E*_a_ = 143 kJ/mol), DOPO-HQ (*E*_a_ = 147 kJ/mol) and DOPO-Van (*E*_a_ = 147 kJ/mol). As the DOPO functional group represents a consistent factor in all additives, the difference in affecting *E*_a_ and the thermal stability of pBF-a was attributed to the aromaticity that increases from DOPO-HQ to DOPO-AP and to DOPO-Van with the highest ratio of phenyl groups.

### 3.3. Determining the Reaction against Small Flames

**Figure 6:**
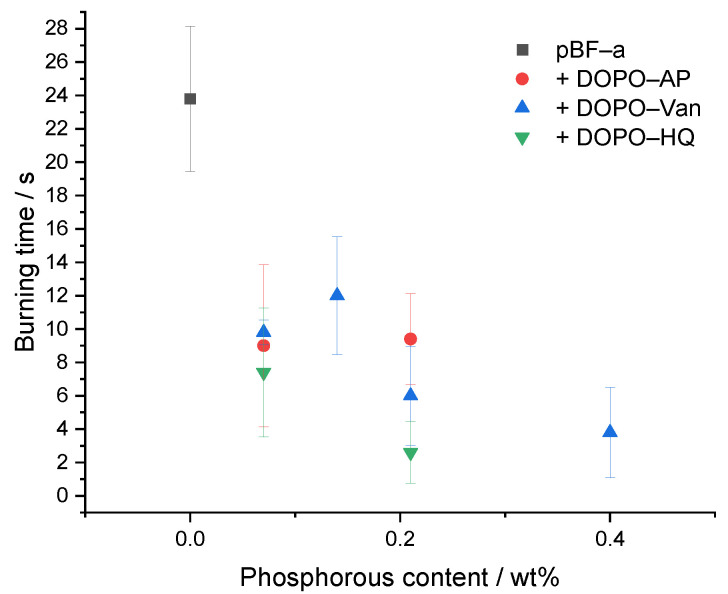
Relation between phosphorous content of different pBF-a mixtures and burning time.

As the pBF-a formulations containing DOPO-based FR additives exhibited an improved thermal stability, the effect on the reaction against small flames was investigated by performing UL-94 vertical burning experiments (Table 2 and Figure 6). Since DOPO-Van contained two DOPO groups, the additional concentrations of 0.5 mol% and 1.5 mol% were chosen to obtain Pwt% formulations that were similar between the three additives. In comparison to neat pBF-a with a V-1 rating, the addition of DOPO-HQ, DOPO-Van and DOPO-AP significantly improved the flame retardancy. With a concentration of 3 mol% DOPO-AP corresponding to a phosphorous content of 0.21 Pwt%, the burning time of the corresponding pBF-a equaled 9.4 s; 3 mol% of DOPO-Van corresponded to 0.4 Pwt% concentration that resulted in 3.8 s of burning time. Thus, with a twice as high amount of Pwt% in DOPO-Van containing benzoxazine samples, the burning time was reduced accordingly. However, 3 mol% DOPO-HQ with a phosphorous content corresponding to 0.21 Pwt% resulted in the shortest burning time with 2.6 s. Degradation temperature and *E*_a_ proved to be slightly lower for DOPO-HQ in facilitating and accelerating the generation of flame retardant volatile radical scavengers that inhibit the flaming combustion process. Except for 1 mol% of DOPO-Van, a V-0 rating for pBF-a can be achieved with a phosphorous content below 0.4 wt% for all additives. Thus, in comparison to other thermosetting polymers such as epoxies, small amounts of additives are sufficient to cause a significant effect to achieve a V-0 rating [37].

**Figure 7:**
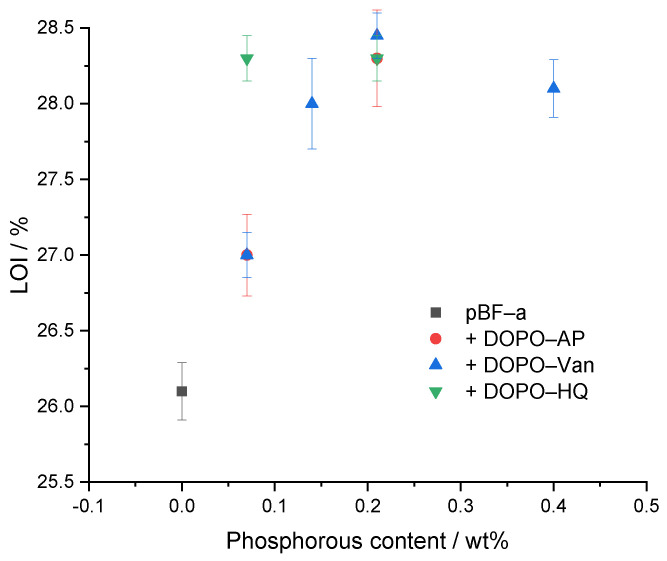
Relation between phosphorous content of pBF-a and FR mixtures and LOI value.

The limited oxygen index (LOI) experiment was performed to determine the ignition behavior (Table 2 and Figure 7). As expected, neat pBF-a had the lowest LOI with 26.1 ± 0.2%. All concentrations of additives improved the LOI value. With all studied DOPO derivatives, a maximum LOI of 28.3% was possible to reach. The responsible phosphorous content, however, varied with 0.07 Pwt% in DOPO-HQ, 0.14 Pwt% in DOPO-Van and 0.21 Pwt% in DOPO-AP. Nevertheless, an increased FR concentration for DOPO-HQ and DOPO-Van did not result in a higher LOI value. DOPO-HQ showed a slightly higher effectivity in comparison to DOPO-Van and DOPO-AP, as less energy is needed to release the radical scavengers.

## 4. Conclusions

The effect of different DOPO derivatives as reactive FR additives in BF-a, namely DOPO-HQ, DOPO-AP and DOPO-Van, on the polymerization, thermo-mechanical properties, and the reaction against small flames, was investigated. The combination of hydroxyl groups and amines proved to decrease ring-opening polymerization temperatures without affecting the overall material properties significantly, in case of DOPO-AP and DOPO-Van. The latter resulted in the strongest effect on BF-a polymerization with a temperature reduction to 198 °C at a concentration of 3 mol%. DOPO-HQ did not significantly affect the polymerization onset, due to the sterical hindrance of the hydroxyl groups.

Despite an increasing concentration and the molecular size of the additives, starting from DOPO-HQ as the smallest molecule, to DOPO-AP and DOPO-Van as the largest, the impact on the network was marginal with only a slight decrease in *T*_g_.

No additives significantly affected the thermal decomposition under nitrogen and ambient atmosphere as well as the a char yield formation at 800 °C. However, the additives proved to increase the initial thermal stability, as a higher *E*_a_ for the decomposition was required. Most of the studied FR additive-containing pBF-a samples achieved a V-0 rating in the UL-94 test, and caused a significant increase in the LOI, up to approximately 28.5%. DOPO-HQ-containing samples, however, improved the reaction against small flames at a very low phosphorous content, while simultaneously maintaining thermo-mechanical stability.

Thus, even small concentrations of halogen-free FR additive beneficially impacted polybenzoxazine polymerization and its final properties, saving resources and protecting the environment.

## Figures and Tables

**Table 1:** TGA test results of pBF-a and FR mixtures under nitrogen atmosphere.

Sample (Composition)	Quantity [mol%]	^a^*T*_1st_ [°C]	^b^ *T*_2nd_ [°C]	^c^ *T*_3rd_ [°C]	^d^ *Y*_c_ [%]
pBF-a		304	401	472	44
+ DOPO-AP	1	302	401	468	47
+ DOPO-AP	3	305	399	467	46
+ DOPO-Van	0.5	298	401	473	43
+ DOPO-Van	1	287	398	472	50
+ DOPO-Van	1.5	288	403	467	43
+ DOPO-Van	3	303	401	464	46
+ DOPO-HQ	1	295	397	464	44
+ DOPO-HQ	3	292	394	468	45

^a^*T*_1st_ temperature at the peak of the first degradation step; ^b^ *T*_2nd_ temperature at the peak of the second degradation step; ^c^ *T*_3rd_ temperature at the peak of the third degradation step, each degradation step temperature was determined by the maximum of the weight-loss derivative, respectively; ^d^ *Y*_c_ residual mass recorded at 800 °C.

**Table 2:** Results for the UL-94 and LOI tests for the pBF-a and FR mixtures.

Additive	Quantity [mol%]	^a^ Pwt%	Total Burning Time [s]	UL-94 Grade	LOI [%]
pBF-a		0	23.8 ± 4.4	V-1	26.1 ± 0.2
DOPO-AP	1	0.07	9.0 ± 4.9	V-0	27.0 ± 0.3
DOPO-AP	3	0.21	9.4 ± 2.7	V-0	28.3 ± 0.3
DOPO-Van	0.5	0.07	9.8 ± 0.7	V-0	27.0 ± 0.2
DOPO-Van	1	0.14	12 ± 3.5	V-1	28.0 ± 0.3
DOPO-Van	1.5	0.21	6.0 ± 3.0	V-0	28.5 ± 0.2
DOPO-Van	3	0.4	3.8 ± 2.7	V-0	28.1 ± 0.2
DOPO-HQ	1	0.07	7.4 ± 3.8	V-0	28.3 ± 0.2
DOPO-HQ	3	0.21	2.6 ± 1.9	V-0	28.3 ± 0.2

^a^ Pw% was calculated from total sample weight.

## Data Availability

Not applicable.

## Supplementary Materials

The following are available online at https://www.mdpi.com/article/10.3390/polym14030606/s1, Video S1. Burning test of pBF-a (a) and mixtures with 3 mol% of DOPO-AP (b), DOPO-HQ (c) and DOPO-Van (d). Table S1: DSC results of BF-a and FR mixtures. Table S2: Test results for DMA experiments. Table S3: TGA test results of pBF-a and FR mixtures under nitrogen atmosphere. Table S4: Results of thermo-oxidative decomposition of pBF-a and FR additives. Table S5: TGA test results of pBF-a and FR mixtures under ambient atmosphere. Figure S1: DSC comparison between BF-a and different concentrations of the corresponding additive DOPO-AP (a), DOPO-Van (b), DOPO-HQ (c). Figure S2: Storage modulus of cured pBF-a and reactive mixtures. Figure S3: Thermal decomposition of pBF-a an FR mixtures. Figure S4: Comparison between thermal decomposition of pBF-a and different concentrations of the FR additives DOPO-AP (a)), DOPO-Van (b) and DOPO-HQ (c). Figure S5: Thermo-oxidative decomposition of pBF-a and mixtures with 3 mol% FR. Figure S6: Comparison between results of thermo-oxidative decomposition pBF-a and different concentrations of the FR additives DOPO-AP (a)), DOPO-Van (b) and DOPO-HQ (c). Figure S7: TGA thermograms of pBF-a (a) and mixturse with 3 mol% of DOPO-AP (b), DOPO-Van (c) and DOPO-HQ (d), respectively, at different heating rates (2, 5, 10, 20 and 40 K/min). Figure S8. Flynn–Wall–Ozawa isoconversion plot for the calculation of activation energy for pBF-a (a) and mixturse with 3 mol% of DOPO-AP (b), DOPO-Van (c) and DOPO-HQ (d), respectively. Figure S9: ^1^H NMR of DOPO-Van. Figure S10: ^31^P NMR of DOPO-Van. Figure S11: FTIR spectra of DOPO-Van. Figure S12: ^1^H NMR of DOPO-HQ. Figure S13: ^31^P NMR of DOPO-HQ. Figure S14: FTIR spectra of DOPO-HQ. Figure S15: ^1^H NMR of 1. Figure S16: ^1^H NMR of DOPO-AP. Figure S17: ^31^P NMR of DOPO-AP. Figure S18: FTIR spectra of DOPO-AP.
