# Supplementary material for: Synthesis and Application Studies of DOPO-Based Organophosphorous Derivatives to Modify the Thermal Behavior of Polybenzoxazine
Source: Polymers (Basel). 2022 Feb 3;14(3):606. doi: 10.3390/polym14030606 (PMC8840307; doi:10.3390/polym14030606)
Supplement: Supplementary file 1 [file polymers-14-00606-s001.zip › polymers-1491539-supplementary.pdf]

Supporting Information

Synthesis and Application Studies of DOPO-Based Organophosphorous Derivatives to Modify the Thermal Behavior of Polybenzoxazine

Thorben Sören Haubold[1,2], Andreas Hartwig[1,2], Katharina Koschek[1*]

[1] Fraunhofer Institute for Manufacturing Technology and Advanced Materials IFAM, Wiener Strasse 12, 28359 Bremen, Germany; Thorben.Haubold@ifam.fraunhofer.de (T.S.H.); Andreas.Hartwig@ifam.fraunhofer.de (A.H.)

[2] University of Bremen, Department 2 Biology/Chemistry, Leobener Strasse 7, 28359 Bremen, Germany

* Correspondence: Katharina.Koschek@ifam.fraunhofer.de

Video S1a.mp4 Video S1b.mp4 Video S1c.mp4 Video S1d.mp4

Video S1. Burning test of pBF-a (**a**) and mixtures with 3 mol% of DOPO-AP (**b**), DOPO-HQ (**c**) and DOPO-Van (**d**).

Sample burning videos according to UL94 test. However, the specimen were not according to DIN EN 60695-11-10 and differ in burning time in comparison to the specimen in the manuscript.

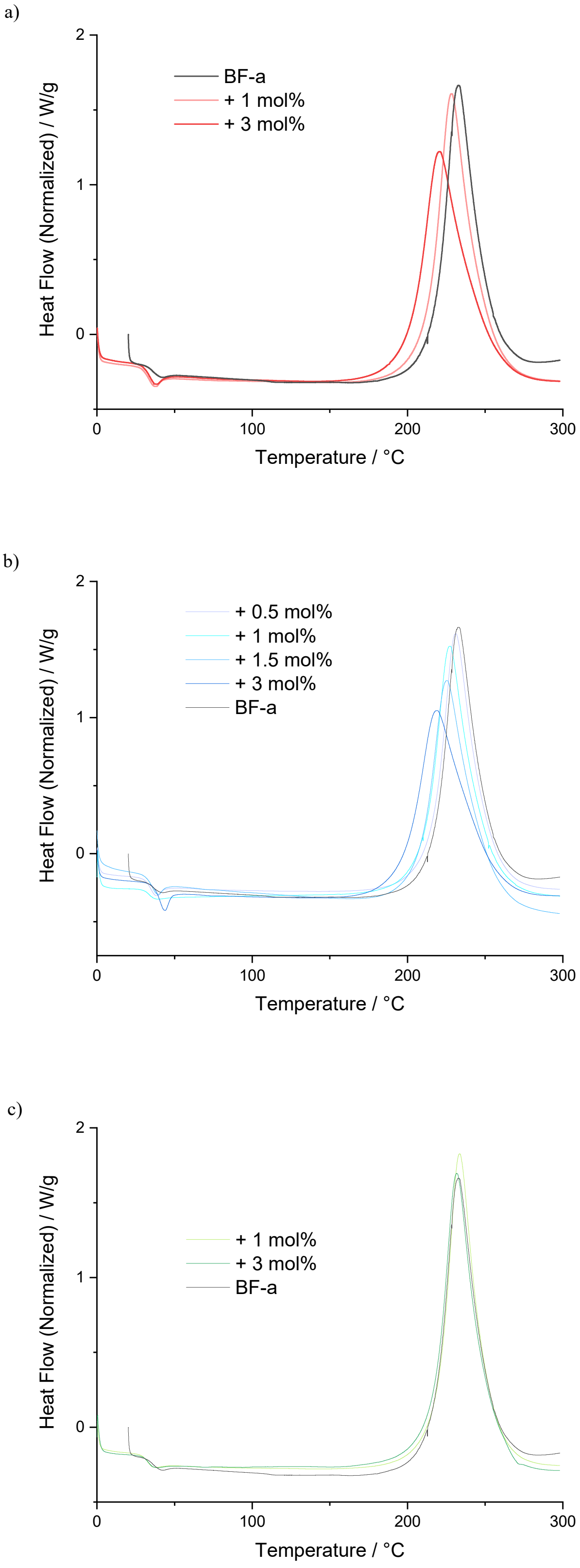

Figure S1. DSC comparison between BF-a and different concentrations of the corresponding additive DOPO-AP (a), DOPO-Van (b), DOPO-HQ (c).

Table S1. DSC results of BF-a and FR mixtures.

Sample (Composition)	Quantity [mol%]	T_{op} [°C]	T_{max} [°C]	ΔH_p [J/g]s	ΔH_{pm} [kJ/mol]
BF-a	0	217	233	290 ± 2	126 ± 1
+ DOPO-AP	1	210	229	302 ± 11	131 ± 5
+ DOPO-AP	3	201	220	315 ± 4	137 ± 2
+ DOPO-Van	0.5	214	230	305 ± 4	133 ± 2
+ DOPO-Van	1	211	228	292 ± 14	128 ± 6
+ DOPO-Van	1.5	207	225	304 ± 3	134 ± 1
+ DOPO-Van	3	198	220	285 ± 10	128 ± 4
+ DOPO-HQ	1	218	233	305 ± 7	132 ± 3
+ DOPO-HQ	3	216	231	297 ± 3	128 ± 1

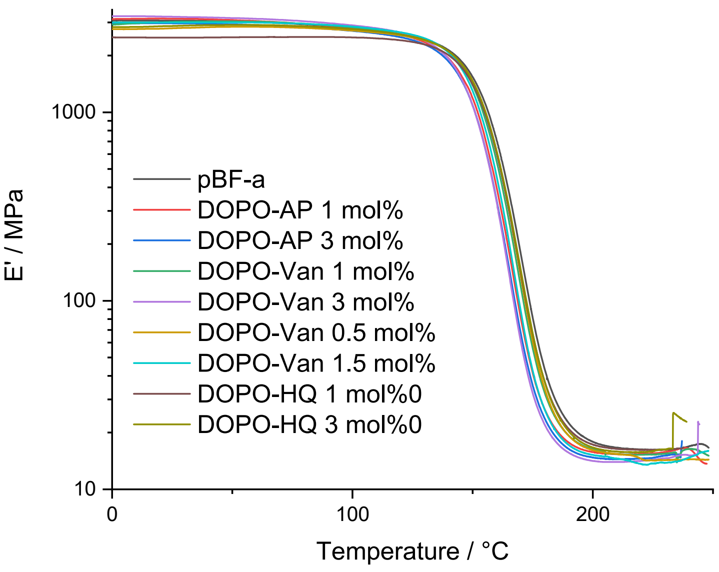

Figure S2. Storage modulus of cured pBF-a and reactive mixtures.

Table S2. Test results for DMA experiments.

Sample (Composition)	Quantity [mol%]	E′ [GPa]	$T_{g\ E''}$ [°C]	$T_{g\ tan(\delta)}$ [°C]
pBF-a		2.8	157	176
+ DOPO-AP	1	2.8	153	172
+ DOPO-AP	3	2.7	153	172
+ DOPO-Van	0.5	2.7	155	174
+ DOPO-Van	1	2.8	151	170
+ DOPO-Van	1.5	2.8	156	174
+ DOPO-Van	3	2.9	153	172
+ DOPO-HQ	1	2.5	156	175
+ DOPO-HQ	3	2.8	155	175

E′ Storage modulus at 100 °C; $T_{g\ E''}$ Glass transition temperature determined by maximum of loss modulus; $T_{g\ tan(\delta)}$ Glass transition temperature determined by the maximum of the tan(δ)

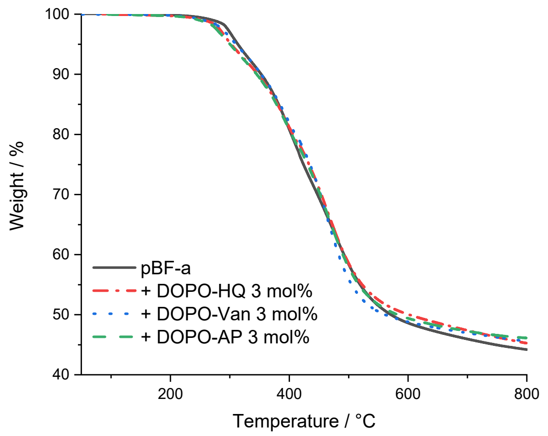

Figure S3. Thermal decomposition of pBF-a an FR mixtures.

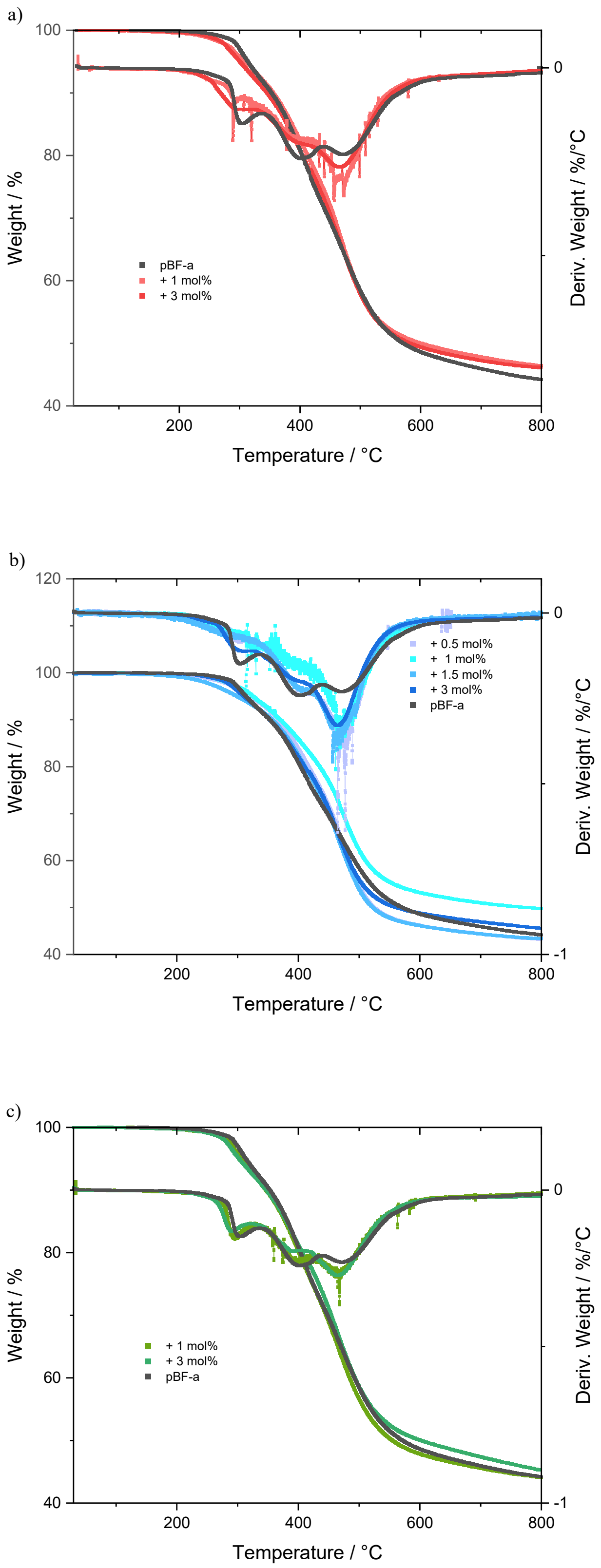

Figure S4. Comparison between thermal decomposition of pBF-a and different concentrations of the FR additives DOPO-AP (a)), DOPO-Van (b) and DOPO-HQ (c).

Table S3. TGA test results of pBF-a and FR mixtures under nitrogen atmosphere.

Sample (Composition)	Quantity [mol%]	$T_{2\%}$ [°C]	$T_{5\%}$ [°C]	$T_{10\%}$ [°C]	$T_{20\%}$ [°C]	Y_c [%]
pBF-a		292	313	352	407	44
+ DOPO-AP	1	271	310	356	412	47
+ DOPO-AP	3	278	306	350	408	46
+ DOPO-Van	0.5	276	321	367	417	43
+ DOPO-Van	1	281	325	370	435	50
+ DOPO-Van	1.5	279	322	362	413	43
+ DOPO-Van	3	286	316	358	410	46
+ DOPO-HQ	1	283	305	345	400	44
+ DOPO-HQ	3	284	308	352	410	45

$T_{2\%}$ temperature at 2% weight loss; $T_{5\%}$ temperature at 5% weight loss; $T_{10\%}$ temperature at 10% weight loss; $T_{20\%}$ temperature at 20% weight loss; Y_c residual mass recorded at 800 °C.

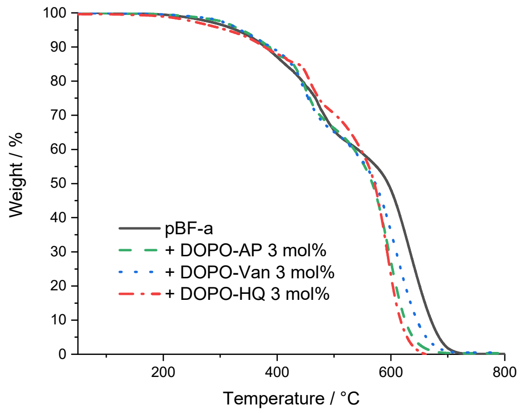

Figure S5. Thermo-oxidative decomposition of pBF-a and mixtures with 3 mol% FR.

a)

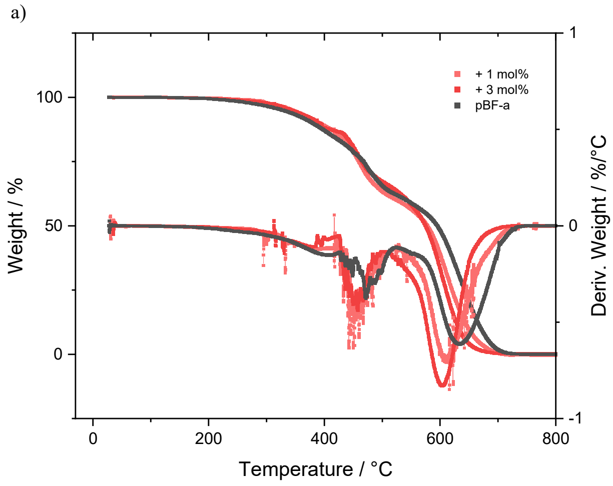

b)

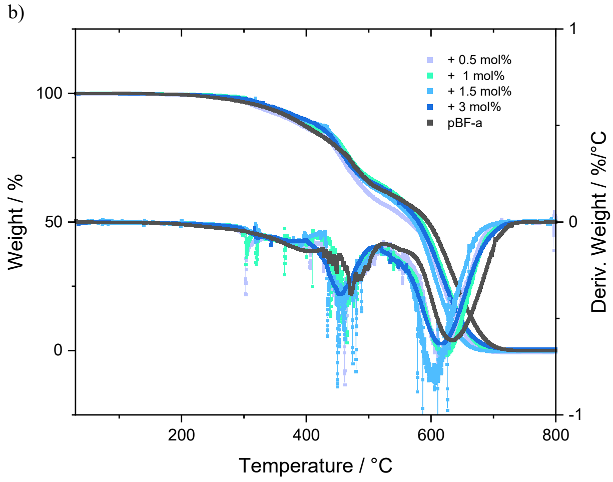

c)

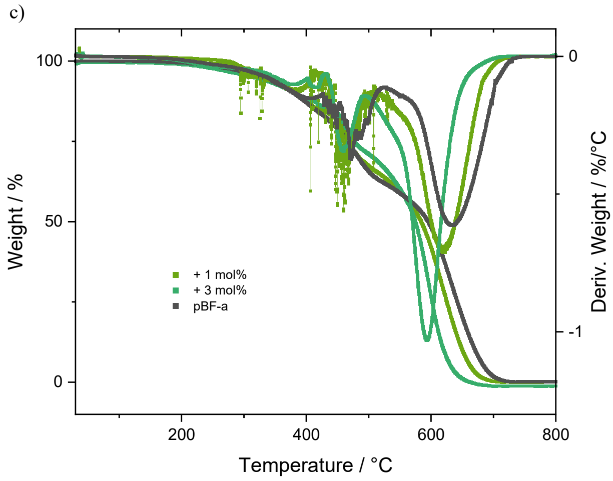

Figure S6. Comparison between results of thermo-oxidative decomposition pBF-a and different concentrations of the FR additives DOPO-AP (a)), DOPO-Van (b) and DOPO-HQ (c).

Tabelle S4. Results of thermo-oxidative decomposition of pBF-a and FR additives.

Sample (Composition)	Quantity [mol%]	T_{1st} [°C]	T_{2nd} [°C]	T_{3rd} [°C]	Y_c [%]
pBF-a		304	401	472	0
+ DOPO-AP	1	302	401	468	0
+ DOPO-AP	3	305	399	467	0
+ DOPO-Van	0.5	298	401	473	0
+ DOPO-Van	1	287	398	472	0
+ DOPO-Van	1.5	288	403	467	0
+ DOPO-Van	3	303	401	464	0
+ DOPO-HQ	1	295	397	464	0
+ DOPO-HQ	3	292	394	468	0

T_{1st} temperature at the peak of the 1st degradation step; T_{2nd} temperature at the peak of the 2nd degradation step; T_{3rd} temperature at the peak of the 3rd degradation step, each degradation step temperature was determined by the maximum of the weight-loss derivative, respectively; Y_c residual mass recorded at 800 °C.

Table S5. TGA test results of pBF-a and FR mixtures under ambient atmosphere.

Sample (Composition)	Quantity [mol%]	$T_{2\%}$ [°C]	$T_{5\%}$ [°C]	$T_{10\%}$ [°C]	Y_c [%]
pBF-a		265	328	380	0
+ DOPO-AP	1	290	345	391	0
+ DOPO-AP	3	294	344	392	0
+ DOPO-Van	0.5	283	319	373	0
+ DOPO-Van	1	303	335	382	0
+ DOPO-Van	1.5	297	337	392	0
+ DOPO-Van	3	299	342	396	0
+ DOPO-HQ	1	298	332	383	0
+ DOPO-HQ	3	277	327	381	0

T_2 temperature at 2% weight loss; T_5 temperature at 5% weight loss; T_{10} temperature at 10% weight loss; Y_c residual mass recorded at 800 °C.

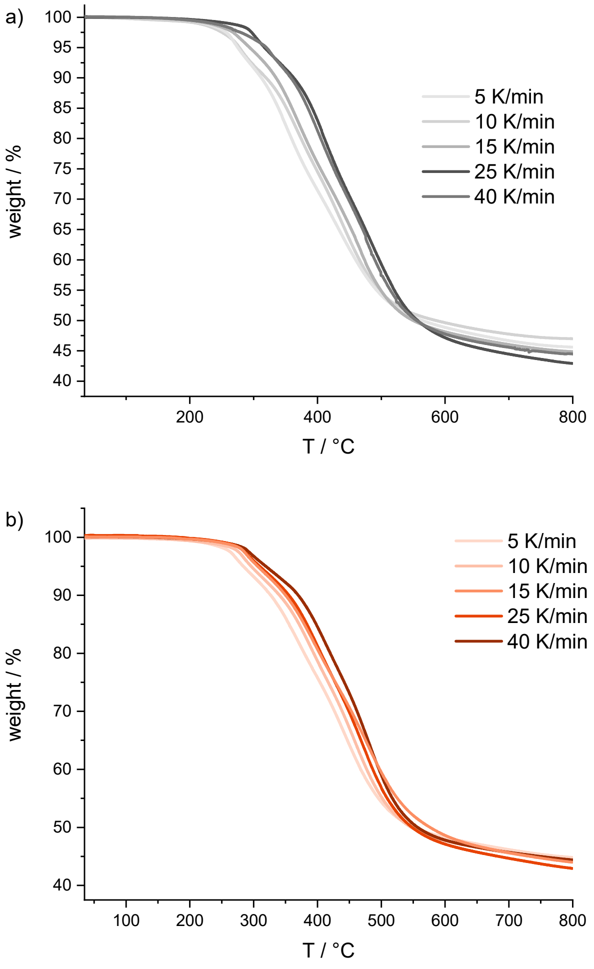

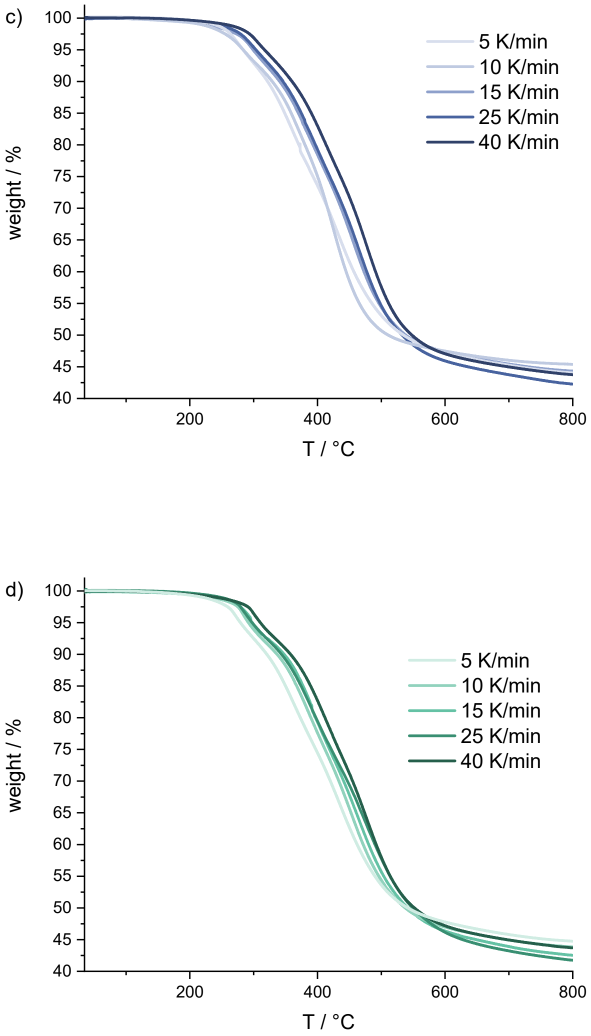

Figure S7. TGA thermograms of pBF-a (**a**) and mixturse with 3 mol% of DOPO-AP (**b**), DOPO-Van (**c**) and DOPO-HQ (**d**), respectively, at different heating rates (2, 5, 10, 20 and 40 K/min).

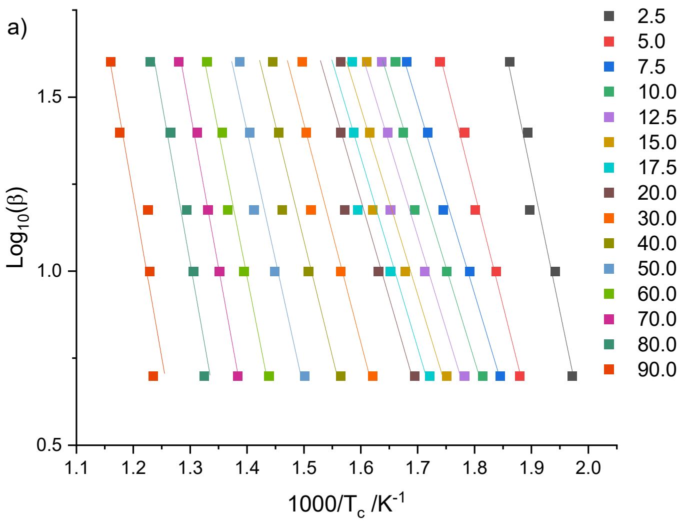
a)
Log10(β)
1000/Tc /K-1
2.5
5.0
7.5
10.0
12.5
15.0
17.5
20.0
30.0
40.0
50.0
60.0
70.0
80.0
90.0

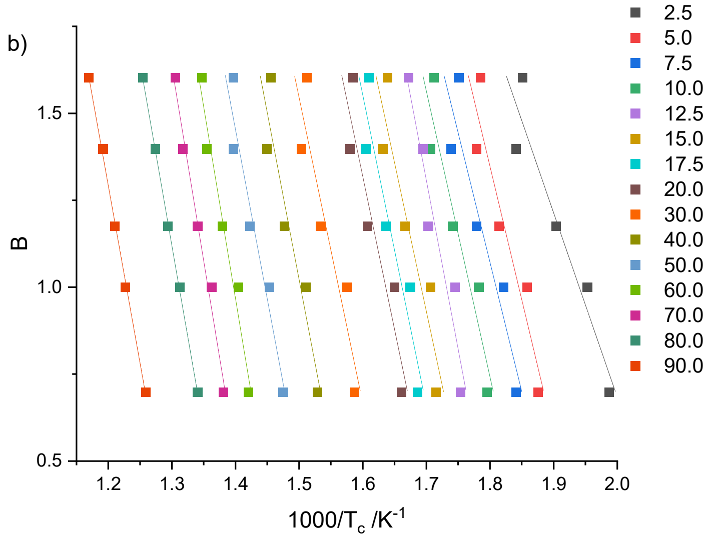
b)
B
1000/Tc /K-1
2.5
5.0
7.5
10.0
12.5
15.0
17.5
20.0
30.0
40.0
50.0
60.0
70.0
80.0
90.0

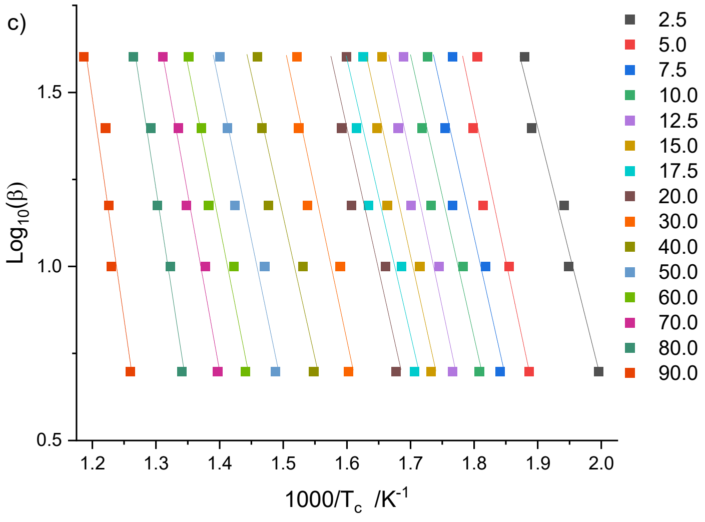
c)
Log10(β)
1000/Tc /K-1
2.5
5.0
7.5
10.0
12.5
15.0
17.5
20.0
30.0
40.0
50.0
60.0
70.0
80.0
90.0

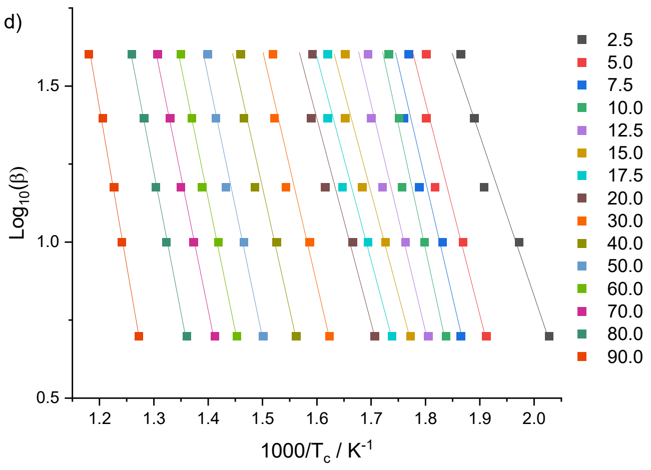

Figure S8. Flynn-Wall-Ozawa isoconversion plot with different heating reats for the calculation of activation energy for pBF-a (**a**) and mixturse with 3 mol% of DOPO-AP (**b**), DOPO-Van (**c**) and DOPO-HQ (**d**), respectively.

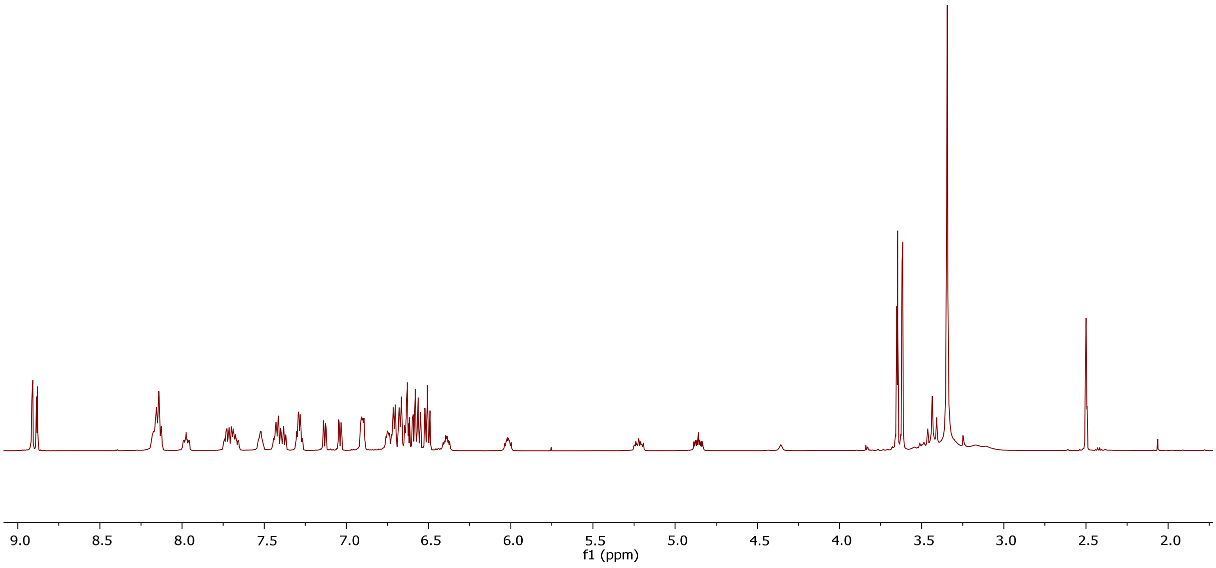

Figure S9. ^{1}H NMR of DOPO-Van.

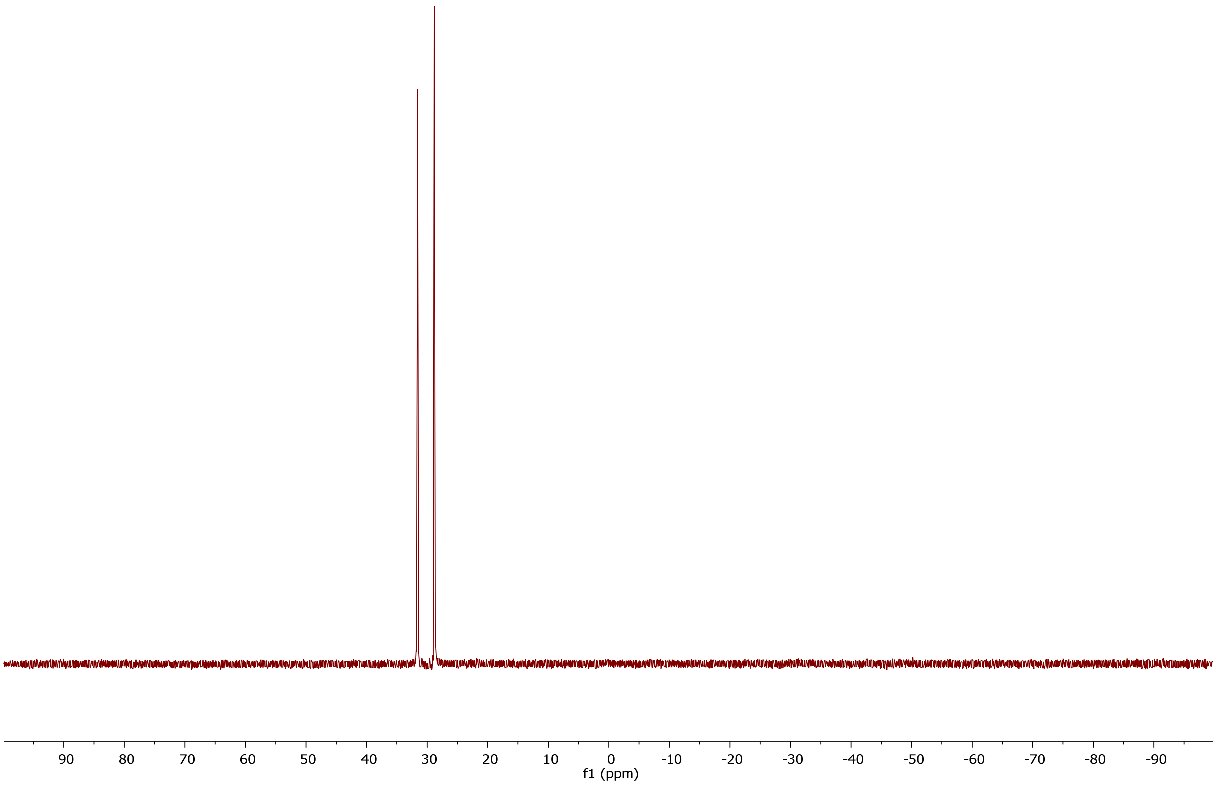

Figure S10. ^{31}P NMR of DOPO-Van.

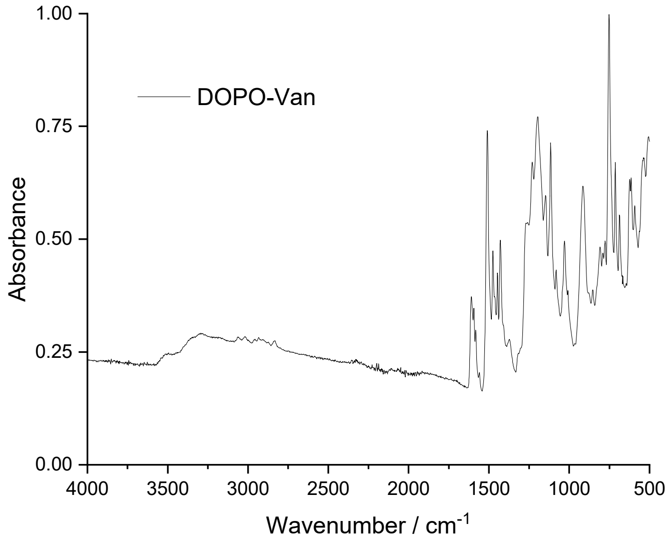

Figure S11. FTIR spectra of DOPO-Van.

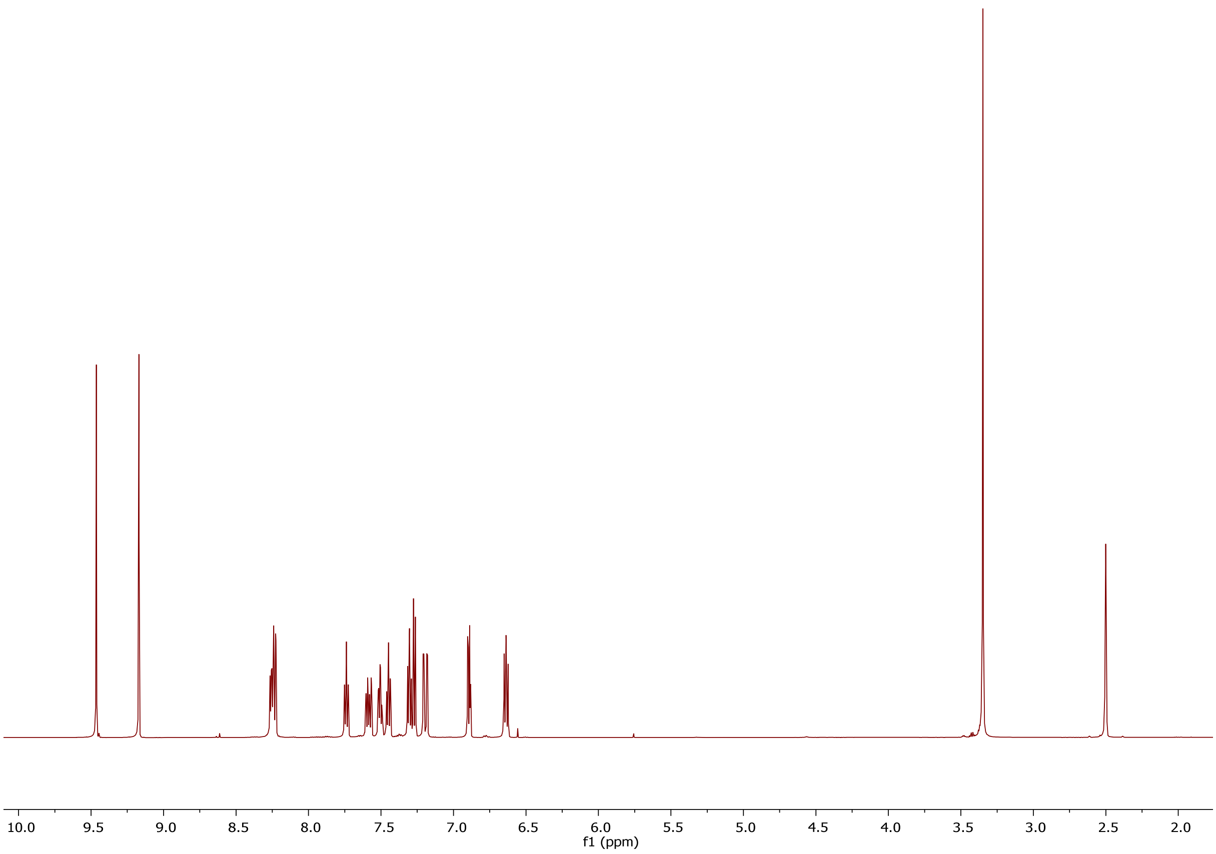

Figure S12. ^{1}H NMR of DOPO-HQ.

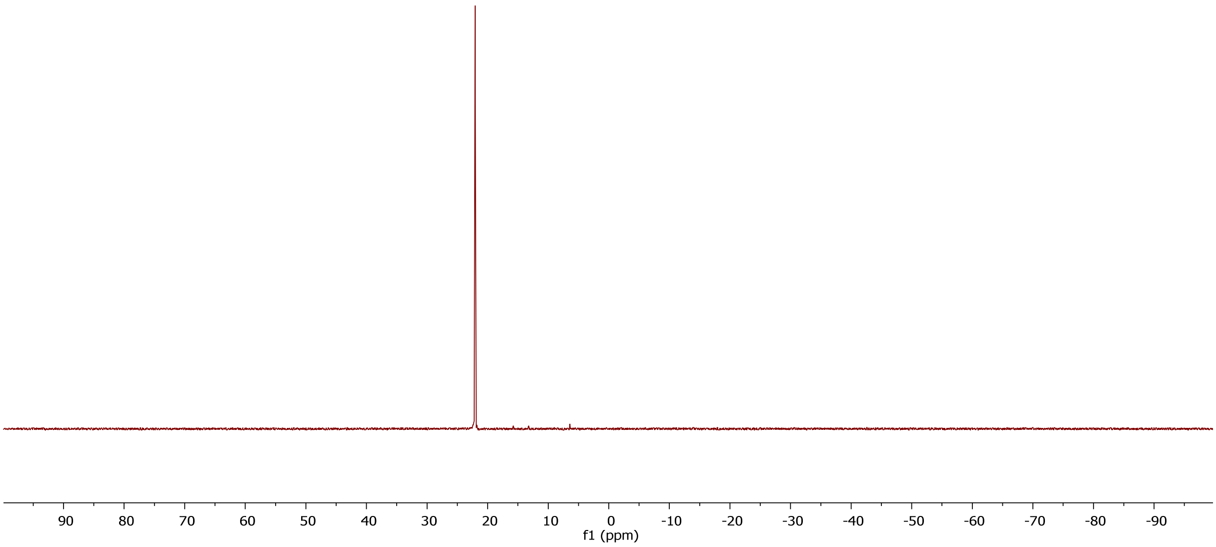

Figure S13. ^{31}P NMR of DOPO-HQ.

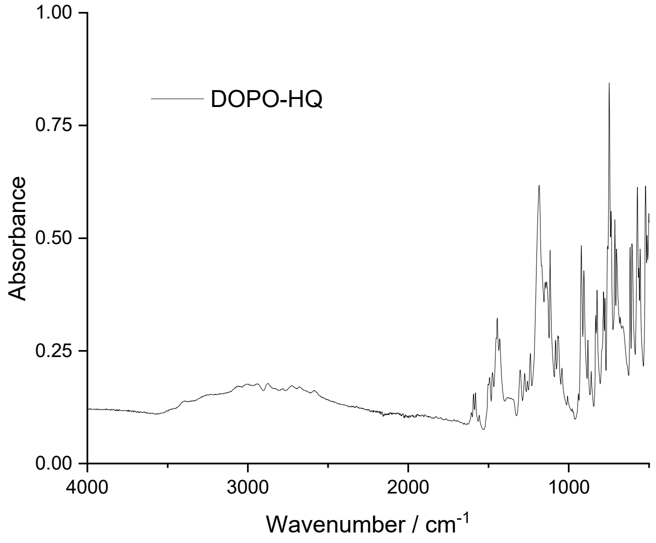

Figure S14. FTIR spectra of DOPO-HQ.

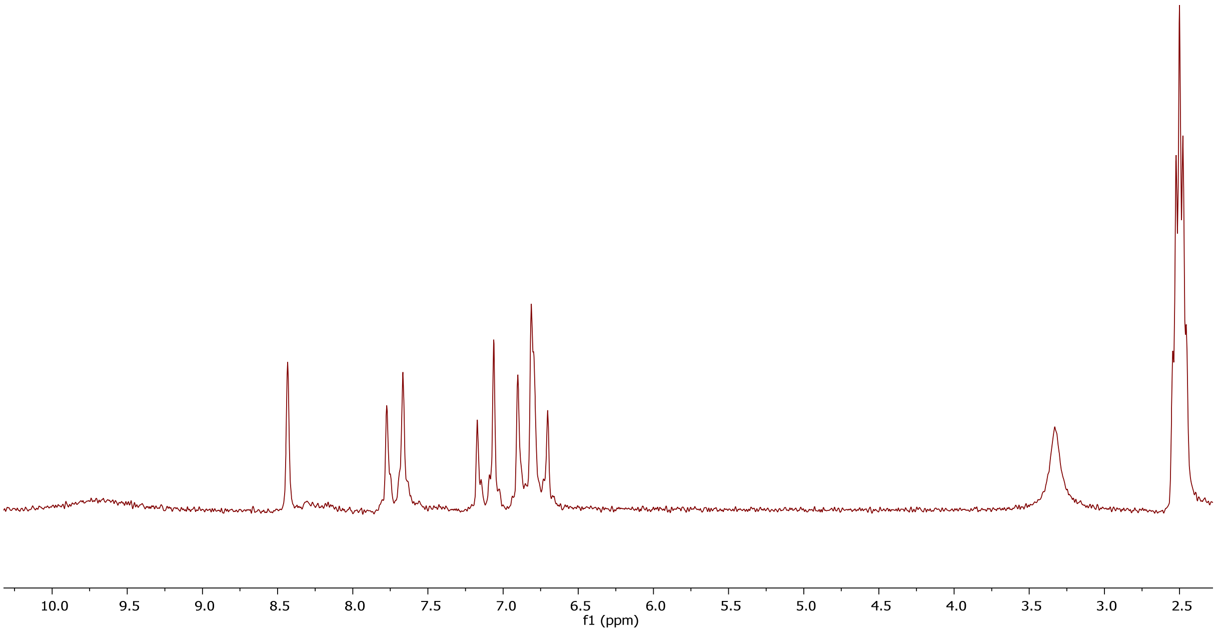

Figure S15. ^{1}H NMR of **1**.

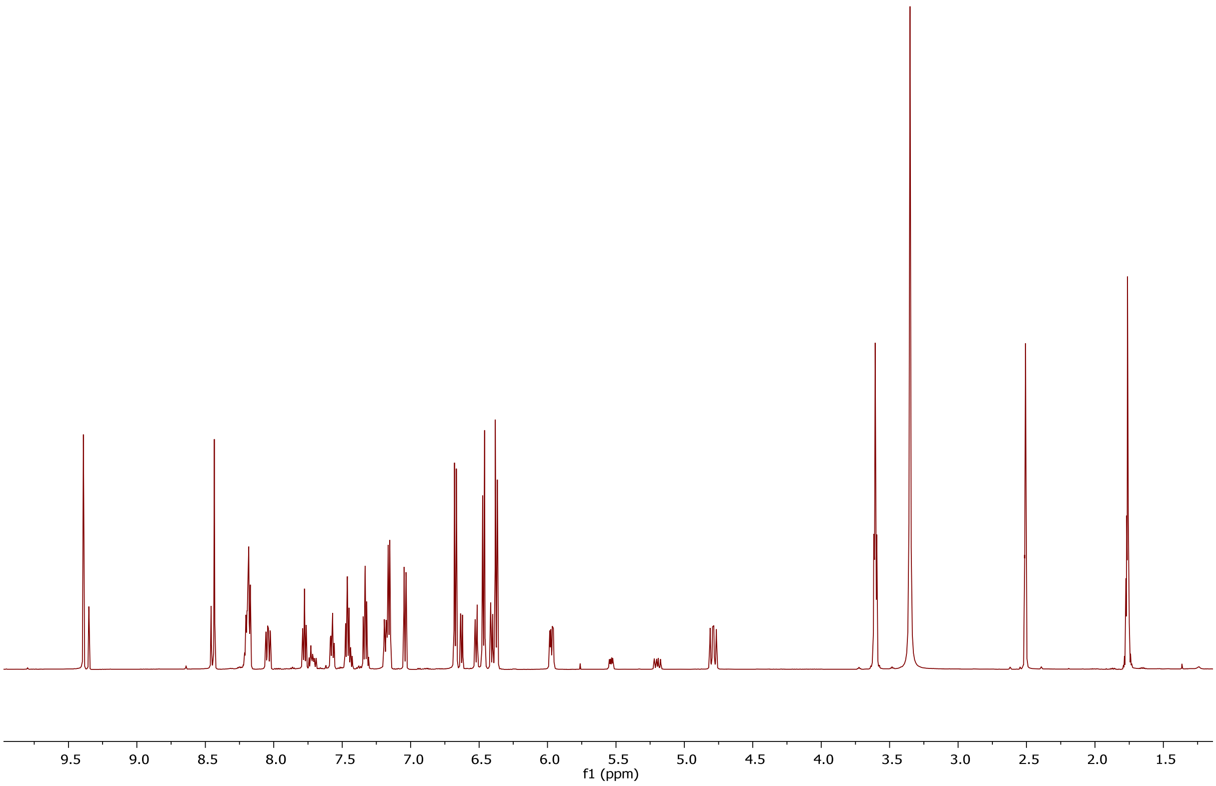

Figure S16. ^{1}H NMR of DOPO-AP.

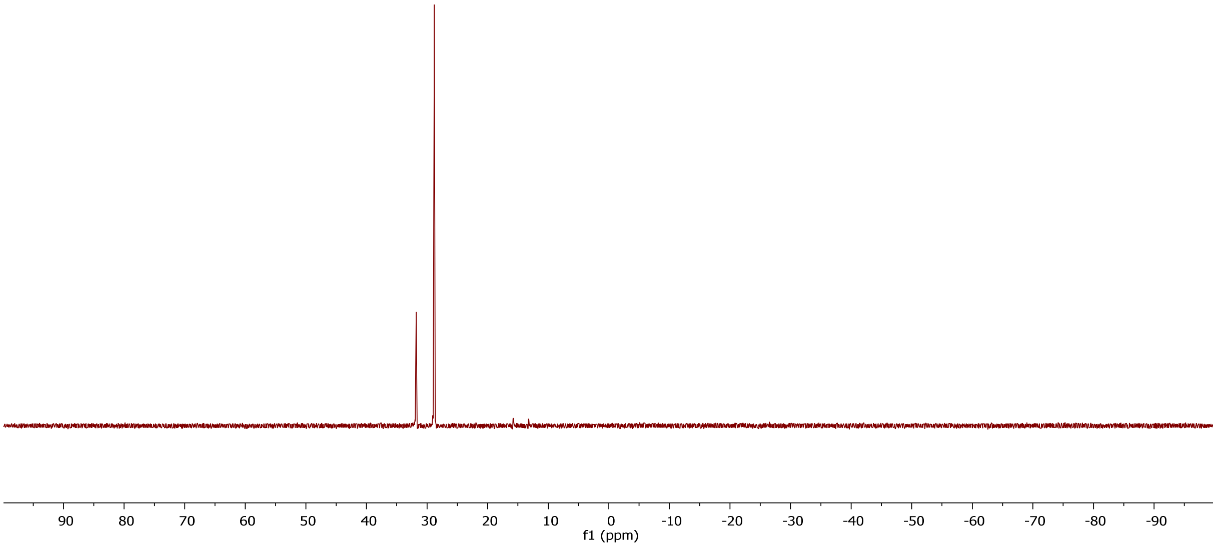

Figure S17. ^{31}P NMR of DOPO-AP.

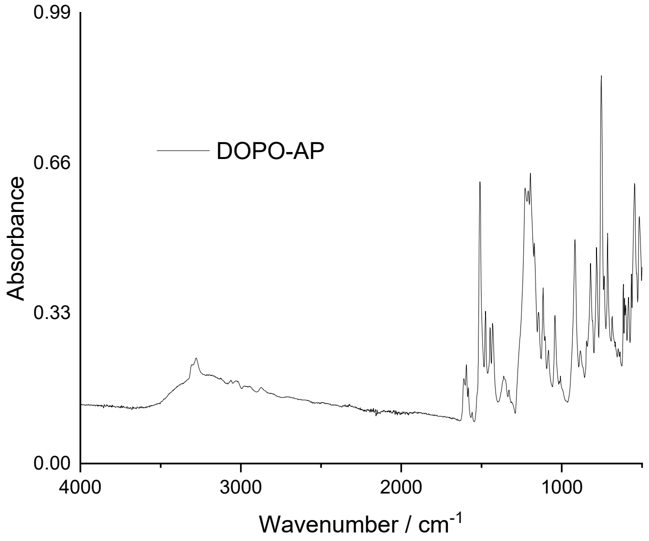

Figure S18. FTIR spectra of DOPO-AP.
